# Community health assessment: Knowledge, attitude and practice of women regarding water-pipe smoking in Bandar Abbas

**DOI:** 10.1016/j.mex.2019.02.026

**Published:** 2019-02-28

**Authors:** Monireh Faghir Ganji, Esrafil Asgari, Maryam Jabbari, Shahrzad Nematollahi, Mona Hosseini, Hassan Ahmadi-Gharaei, Ali ArabAhmadi, Mohsen Ostad Ghaderi, Kourosh Holakouie-Naieni

**Affiliations:** a Department of Epidemiology and Biostatistics, School of Public Health, Tehran University of Medical Sciences, Tehran, Iran; b Department of Environmental Health Engineering, Khoy University of Medical Sciences, Khoy, Iran; c Department of Public Health, School of Paramedical and Health, Zanjan University of Medical Sciences, Zanjan, Iran; d Department of Environmental Health Engineering, School of Health, Isfahan University of Medical Sciences, Isfahan, Iran

**Keywords:** Educational models, Community health assessment, Diagnosis, Smoking water-pipe, Knowledge, Attitude, Practice, Bandar Abbas

## Abstract

Based on the results of seventh round of community health assessment (CHA) in suburban areas of Bandar Abbas, "water-pipe smoking in women" was one of the major concerns of community members. Therefore, the present study designed to assess the knowledge, attitude and practice of women towards water-pipe smoking and related factors. High consumption of water-pipe among women was ranked as a prioritized health problem. To diagnose the problem, for creating action plan, the present cross - sectional study was conducted on 205 women aged over 18 randomly selected from Green-Tree region in suburb of Bandar Abbas city. All statistical analyses were performed using SPSS 24 software with 5% as the significant level. 205 women with a mean age of 36.9 (standard deviation:12.86) years, and a water-pipe prevalence of 15.1% were analyzed. The significant predictors of knowledge were educational level (β = 0.182, p-value = 0.037), and being water-pipe smoker (β = −0.251, p-value < 0.001); while for attitude they were educational level (β = 0.221, p-value = 0.002), family size(β = 0.152, p-value = 0.023), and subjective social status(β = 0.149; p-value = 0.035); and for practice they was smoking waterpipe in parents (β = −0.276, p-value < 0.001).The development action plans based on “CHA” could improve public health and enhance the performance of the community through improved education, policies and health interventions.

## Graphical abstract

**Figure:**
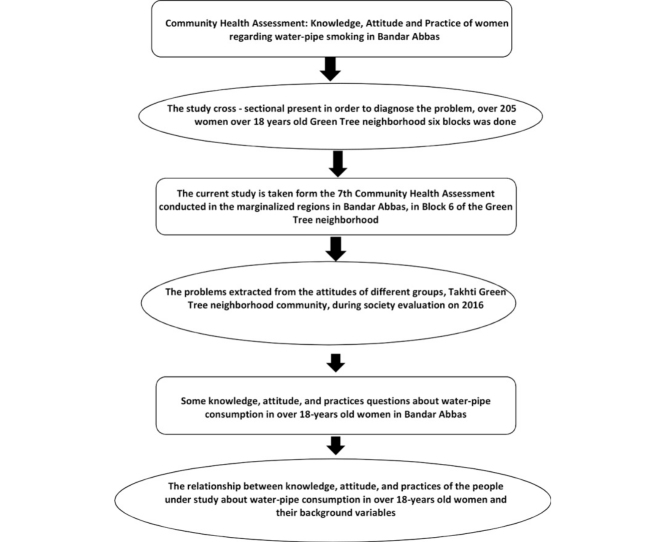

**Specifications Table**

**Table:** 

**Subject area**:	*Epidemiology*
**More specific subject area**:	Community Health Assessment
**Protocol name**:	Application of Community Health Assessment: Knowledge, Attitude and Practice of women regarding water-pipe smoking
**Reagents/tools**:	The current study is taken form the 7th Community Health Assessment conducted in the marginalized regions in Bandar Abbas, in Block 6 of the Green Tree neighborhood.
**Experimental design**:	To diagnose the problem, for creating action plan, the present cross sectional study was conducted on 205 women aged over 18 randomly selected from Green-tree region in suburb of Bandar Abbas city
**Trial registration**:	No applicable
**Ethics**:	No applicable

## Protocol data

• Community health assessment with public participation can provide better understanding the needs of society, researchers and health professionals [[1], [2], [3], [4], [5]].
• The assessment results showed that community health concerns and priorities of the community members may be different from what health systems report.
• Operational plans based on diagnostic problems in the community, for education, policy and health interventions could improve public health and enhance the performance of people.
• The implementation of the public health assessment process, leading to increased participation of people in their health and prevention programs are guaranteed to run properly.

## Description of protocol

### Materials and methods

This study is conducted in the form of a community evaluation project in Block 6 of Green Tree neighborhood in the north eastern part of Bandar Abbas City, Hormozgan Province. The model used in this study is based on the community evaluation pattern used in the School of Public Health, Tehran University of Medical Sciences [6,7], to evaluate different communities based on the localized pattern [8] of Evaluation of the North Carolina Health Administration Society [[9], [10], [11]].

According to this pattern, community evaluation consists of eight phases, in which phases one to seven contribute to problem identification and prioritization, and phase eight deals with developing an operational plan to solve the problems. In phase one, the community evaluation team, consisted of one epidemiologist as the guide, one person responsible for coordinating with the administrations and residents in the region, and two secretaries for interview meetings. The primary data were collected in the second phase during group focused discussion sessions, brain storming and individual interviewing in Shahid Takhty's Health Center, the schools and mosques in the neighborhood, teachers, residents and trusties in the region.

Data gathering, summarizing and interpreting the primary and secondary data obtained from other organizations was done in the third phase. Analyzing the data obtained from phases two and three, aiming to acquire a fundamental understanding of the demographic features, major health risks and the existing problems was done in the fourth phase, and totally 67 problems were extracted as a result. Oral and written report of the evaluation process of the beneficiaries in society was done in the fifth phase to involving more people in the evaluation process. In the sixth phase, prioritizing the problems identified in the previous phases was done in a collaborative meeting, in presence of the evaluation team, health professionals, the mosque liturgist, board of trustees, and the trusties in the neighborhood, using Hanlon method [12].

Scoring the problems listed based on health importance, the extent and feasibility of addressing the problem was done by assigning 1–10 to each problem, and the first 10 problems, having the highest score, were selected as the high priority problems in the community under study (Table 1).

**Table 1:** The problems extracted from the attitudes of different groups, Takhti Green Tree neighborhood community, during society evaluation in 2016.

No.	Health Authorities	People in the Neighborhood	Final Prioritization
1	Water and Wastewater	Youth unemployment	Youth unemployment
2	Lack of proper waste collection and lack of trash bin	Addiction	Presence of mice and rodents
3	Youth unemployment	Lack of proper waste collection and lack of trash bin	High prevalence of addiction and men selling drugs
4	Domestic violence	Outbreak of head lice	Economic and cultural poverty and divorce
5	Easy access to drugs and addiction	High consumption of water-pipe in women	Outbreak of head lice
6	High prevalence of vitamin D deficiency	High consumption of water-pipe in women	Alcohol consumption and abuse
7	Divorce	Presence of mice and rodents	Problems in waste collection
8	Economic and cultural poverty	High prevalence of anxiety	Mothers’ low knowledge about the use of supplements for their children
9	Rusty houses	Low knowledge about the use of supplements	Lack of suitable sports space
10	High immigration	Easy access to drugs	Water-pipe abuse in women

Finally, the problem "high prevalence of water-pipe in women" was selected among the list, which had operational, educational, and intervention feasibility. The current field study, aiming to identify this problem in Block 6 of Green Tree neighborhood in Bandar Abbas was conducted by developing a proposal entitled "Investigating the knowledge, attitude, and practices of over 18-years old women living in Block 6 of Green Tree neighborhood about using water-pipe and the factors affecting it, aiming to develop an operational plan in 2016″.

Documentary report on the evaluation process, along with all findings, to the society members is done in the seventh phase, which, in turn, results in developing an operational plan for the society in the eighth phase for high priority problems. To run this cross-sectional study, 206 women over 18-years old, residing in Block 6 of Green Tree neighborhood, were selected randomly (Table 2).

**Table 2:** Some knowledge, attitude, and practices questions about water-pipe consumption in over 18-years old women in Bandar Abbas.

	Correct Answer	
Scope of knowledge	Frequency	Percent
Does water-pipe contains a lot of poisonous materials?	178	86.6
Does Water-pipe induce less addiction compared to cigarette?	46	22.4
Does smoking water-pipe lead to cardiovascular problems?	192	93.7
Scope of attitude		
In my opinion, addiction to water-pipe has no danger	94	45.9
In my opinion, passing the water-pipe smoke through water removes its toxic materials.	55	26.8
In my opinion, water-pipe consumption is more approved in the society compared to cigarettes.	22	10.7
In my opinion, water-pipe consumption decreases anxiety.	44	21.5
Scope of practices		
I have the experience of giving up water-pipe consumption.	25	12.2
I gave up water-pipe because of my parents' opposition.	185	90.2
I have personal water-pipe.	21	10.2
Access to water-pipe is easy for me.	24	11.7
I consulted on the dangers of water-pipe consumption.	190	92.7

The study used the researcher-made survey questionnaire as a principal tool for gathering data. The questionnaire included 5 sections: demographic information, knowledge, attitude, practices, and life satisfaction questions [13,14]. The questioners completed the questionnaire. The content validity of the questionnaire was verified using the experts’ attitude, and the questions’ reliability in each field was verified using the primary studies (Cronbach's alpha = 0.79).

The data analysis was performed in two descriptive and analytical parts. Mean value, standard deviation, and relative frequency indices were used in the descriptive part, while Chi-square test and linear regression models were used in the analytical part. The outcome variables in this study (knowledge, attitude, and practices) were divided from the mean value to two parts: higher than mean and lower than mean (Table 3); two-state variables were used as outcome for further analysis. Statistical analyses were done using SPSS software, version 24.

**Table 3:** The relationship between knowledge, attitude, and practices of the people under study about water-pipe consumption in over 18-years old women and their background variables.

			Knowledge			Attitude			Practices		
Variable name	Variable levels	Frequency	Frequency of people higher than mean value	Percent of people higher than mean value	Statistical test	Frequency of people higher than mean value	Percent of people higher than mean value	Statistical test	Frequency of people higher than mean value	Percent of people higher than mean value	Statistical test
Age	> 36	120	85	70.8	$\chi^{2}$ = 0.68	71	59.2	$\chi^{2}$ = 1.48	102	85	$\chi^{2}$ = 0.003
	≤ 36	85	53	62.4	P = 0.2	43	50.6	P=0.2	72	84.7	P = 0.95
Marital Status	Signe	22	17	77.3	$\chi^{2}$ = 3.07	8	36.4	$\chi^{2}$ = 13.2	17	77.3	$\chi^{2}$ = 1.43
	Married	165	111	67.3	P = 0.38	101	61.2	P=0.004	142	86.1	P = 0.69
	Divorced	1	1	100		1	100		1	100	
	Widow	17	9	52.9		4	23.5		14	82.4	
The last academic degree earned	Illiterate	29	16	55.2	$\chi^{2}$ = 8.98	10	34.5	$\chi^{2}$ = 8.42	24	82.8	$\chi^{2}$ = 0.85
	Primary school	54	33	61.1	P = 0.061	28	51.9	P = 0.07	46	85.2	P = 0.93
	Secondary school	37	27	73		21	56.8		30	81.1	
	Diploma	68	46	67.6		44	64.7		59	86.8	
	University	17	16	94.1		11	64.7		15	88.2	
Occupation	Housewife	169	111	65.7	$\chi^{2}$ = 7.71	96	56.8	$\chi^{2}$=0.94	144	85.2	$\chi^{2}$ = 11.95
	Employee	3	3	100	P = 0.155	1	33.3	P = 0.96	1	33.3	P = 0.03
	Worker	4	1	25		2	50		2	50	
	Self employed	21	16	76.2		11	52.4		19	90	
	Unemployed	4	3	75		2	50		4	100	
	Others	4	4	100		2	50		4	100	
Family Dimension	> 4	82	60	73.2	$\chi^{2}$ = 2.12	45	54.9	$\chi^{2}$= 0.03	69	84.1	$\chi^{2}$ = 0.03
	≤4	123	78	63.4	P = 0.095	69	56.1	P = 0.86	105	85.4	P = 0.86
Family financial status compared to relatives	Poor	41	25	61	$\chi^{2}$= 1.44	17	41.5	$\chi^{2}$ = 6.16	33	80.5	$\chi^{2}$ = 0.93
	Moderate	129	88	68.2	P = 0.073	73	56.6	P = 0.10	111	86	P = 0.81
	Good	34	24	70		23	67.6		29	85.3	
	Rich	1	1	100		1	100		1	100	
Individual health status	Very bad	3	3	100	$\chi^{2}$ = 6.06	0	0	$\chi^{2}$ = 6.81	2	66.7	$\chi^{2}$ = 1
	Bad	11	8	72.7	P = 0.191	4	36.4	P = 0.14	9	81.8	P = 0.90
	Moderate	61	36	59		34	55.7		52	85.2	
	Good	125	86	68.8		72	57.6		107	85.6	
	Very Good	5	5	100	≤	4	80		4	80	
Parents smoking water-pipe or cigarette	No	156	109	69.9	$\chi^{2}$ = 1.93	91	58.3	$\chi^{2}$ = 1.96	140	89.7	$\chi^{2}$ = 12.03
	Yes	49	29	59.2	P = 0.221	23	46.9	P = 0.16	34	69.4	P < 0.001

## Conflicts of interest

Authors have no conflicts of interest.
